# Low Genetic Diversity of the Only Clade of the Tick *Rhipicephalus microplus* in the Neotropics

**DOI:** 10.3390/pathogens12111344

**Published:** 2023-11-13

**Authors:** Sandra Díaz-Sánchez, Luis M. Hernández-Triana, Marcelo B. Labruna, Octavio Merino, Juan Mosqueda, Santiago Nava, Matias Szabó, Evelina Tarragona, José M. Venzal, José de la Fuente, Agustín Estrada-Peña

**Affiliations:** 1 SaBio, Instituto de Investigación en Recursos Cinegéticos IREC-CSIC-UCLM-JCCM, Ronda de Toledo s/n, 13005 Ciudad Real, Spain; sandra.dsan@gmail.com (S.D.-S.); jose_delafuente@yahoo.com (J.d.l.F.); 2 Animal and Plant Health Agency, Virology Department, Surrey KT15 3NB, UK; l.hernandez-triana@vla.defra.gsi.gov.uk; 3 Faculty of Veterinary Medicine, São Paulo 05508-270, SP, Brazil; labruna@usp.br; 4 Faculty of Veterinary Medicine, Universidad Autónoma de Tamaulipas, Tamaulipas 87000, Mexico; mero840125@hotmail.com; 5 Laboratory for Research on Immunology and Vaccines, Facultad de Veterinaria, Querétaro 76230, Mexico; joel.mosqueda@uaq.mx; 6 IDICAL (INTA-CONICET), Instituto Nacional de Tecnología Agropecuaria (INTA), E.E.A. Rafaela, Rafaela 2300, Santa Fe, Argentina; nava.santiago@inta.gob.ar (S.N.); tarragona.evelina@inta.gob.ar (E.T.); 7 Hospital Veterinário, Universidade Federal de Uberlândia, Uberlândia 38405-314, MG, Brazil; szabo@famev.ufu.br; 8 Departamento de Ciencias Biológicas, CENUR Litoral Norte, Universidad de la República, Salto 50000, Uruguay; dpvuru@hotmail.com; 9 Department of Veterinary Pathobiology, Center for Veterinary Health Sciences, Oklahoma State University, Stillwater, OK 74078, USA; 10 Department of Animal Health, Faculty of Veterinary Medicine, 50009 Zaragoza, Spain; 11 Group of Research on Emerging Zoonoses, Instituto Agroalimentario de Aragón (IA2), 50013 Zaragoza, Spain

**Keywords:** *Rhipicephalus microplus*, *COI*, *16S rDNA*, *ITS-2*, Neotropics, clade A, climate traits, evolution rates

## Abstract

This study addresses the variability of the mitochondrial cytochrome oxidase subunit I (*COI*) and *16S rDNA* (*16S*), and nuclear internal transcriber spacer *ITS2* (*ITS2*) genes in a set of field-collected samples of the cattle tick, *Rhipicephalus microplus* (Canestrini, 1888), and in geo-referenced sequences obtained from GenBank. Since the tick is currently considered to be a complex of cryptic taxa in several regions of the world, the main aims of the study are (i) to provide evidence of the clades of the tick present in the Neotropics, (ii) to explore if there is an effect of climate traits on the divergence rates of the target genes, and (iii) to check for a relationship between geographical and genetic distance among populations (the closest, the most similar, meaning for slow spread). We included published sequences of *Rhipicephalus annulatus* (Nearctic, Afrotropical, and Mediterranean) and *R. microplus* (Afrotropical, Indomalayan) to fully characterize the Neotropical populations (total: 74 *16S*, 44 *COI*, and 49 *ITS2* sequences included in the analysis). Only the clade A of *R. microplus* spread in the Nearctic–Neotropics. Both the K and Lambda’s statistics, two measures of phylogenetic signal, support low divergence rates of the tested genes in populations of *R. microplus* in the Neotropics. These tests demonstrate that genetic diversity of the continental populations does not correlate either with the geographic distance among samples or with environmental variables. The low variability of these genes may be due to a combination of factors like (i) the recent introduction of the tick in the Neotropics, (ii) a large, effective, and fast exchange of populations, and (iii) a low effect of climate on the evolution rates of the target genes. These results have implications for the ecological studies and control of cattle tick infestations.

## 1. Introduction

Six *Rhipicephalus (Boophilus)* tick species are currently recognized, according to Guglielmone et al. (2014): *Rhipicephalus annulatus* (Say, 1821), *Rhipicephalus decoloratus* (Koch, 1844), *Rhipicephalus microplus* (Canestrini, 1888), *Rhipicephalus australis* Fuller, 1899, *Rhipicephalus kohlsi* (Hoogstraal and Kaiser, 1960), and *Rhipicephalus geigyi* (Aeschlimann and Morel, 1965). It has been postulated that the ancestral range of these species is the Oriental region [1]. *Rhipicephalus microplus* is a tick that has undergone a significant geographical expansion, including large areas in Asia (its presumed original range), and in a wide range of the Neotropics, and Africa [2].

*Rhipicephalus microplus* is regarded as one of the most serious pests affecting cattle health and production [3]. It is assumed that *R. microplus* was introduced in the Neotropics from and into yet unknown sites, most probably with the cattle trade, probably around the XVII century. It is unknown that the tick was introduced only once, like *Amblyomma variegatum* in the Caribbean islands [4] or had several introductions at different points alongside the region. Currently, the Nearctic–Neotropical range of the cattle tick extends from Southern USA to Argentina, around the latitude 32°S. The southern fringe of *R. microplus* in the Neotropics fluctuates because of the pattern of winter temperature, limiting its spread further south [5].

Based on recent phylogenetic analyses *R. microplus* has been considered as a complex of species [1]. This complex is structured in three clades named clade A or *R. microplus* s.s. originating in southeast Asia [6], clade B with ticks from China and northern India that are closer to *R. annulatus* [6], and clade C from Bangladesh, India, Malaysia, and Pakistan [1,7]. These phylogenies have been obtained using sequences of the mitochondrial cytochrome oxidase subunit I (*COI*) and *16S rDNA (16S)* genes, demonstrating the power that these gene markers can provide to separate cryptic species. For example, the mitochondrial markers (*COI* and *16S)* have been the preferred marker together with the nuclear internal transcriber 2 gene *(ITS2*) marker, to track the divergence of *R. microplus* populations in South Africa [8], western Africa [9] Asia, Brazil [6], and India [10].

Genetic studies including nuclear genes or single nucleotide polymorphisms (SNPs) have been proposed as suitable for resolving specific identities of ticks [11,12]. Individual gene markers, microsatellites, or SNPs have been used for some species of ticks to explore hybridization, mitochondrial introgression, phylogenetic relationships, interspecific variation, and comparison between mitochondrial and nuclear markers for species/lineage delimitation [13,14,15,16,17].

The genetic identity of *R. microplus* in the Neotropics has been never examined [5]. There is no evidence that *R. microplus* in the Neotropics have a clear population divergence; it is unknown if climate patterns in its large Neotropical range may impact the genetic diversification of the tick’s populations. The Neotropics have areas of rapid transition between humid and arid climates likely producing sharp changes in the diversity of tick assemblages and/or the adaptation of *R. microplus* to different environmental conditions. The species fundamental niche is “all the possible combinations of environmental traits where a species can persist and maintain a viable population in the absence of predators or competitors” [18]. The fundamental niche derived from climate patterns is not itself a heritable trait. However, the characteristics of the niche are defined and constrained by species physiology, which is heritable, and as such can be analyzed in a phylogenetic framework [19,20]. 

Phylogenetic niche conservatism (PNC) is the tendency of lineages to retain their ancestral ecological niche through speciation events [21]. This is of special interest in the context of an invasive tick species because it means that the ancestral niche could be reflected in the genetic signature of the tick and modified by local environmental traits. Some of the phylogenetic studies conducted in recent years on different organisms indicated that major aspects of the niche are more preserved during evolution and speciation than expected [22,23].

It has been suggested that genetic features could be related to the ability to spread by alien species [24,25], which immediately suggests the restriction of their spread by biotic (the existence of vertebrate hosts) other than abiotic pressures. Studies have reported a spatial genetic heterogeneity of tick populations colonizing large areas, suggesting local or regional processes of specialization to prevailing climate conditions and/or host availability. In studies in which this issue has been explicitly addressed, the PNC paradigm has supported the existence of partially overlapping ecological ranges of different tick species [26,27]; this could be interpreted as the beginning of a phenomenon of speciation of the organisms colonizing a given gradient of climate variables. 

In this study, we propose to evaluate the populations of *R. microplus* in the Nearctic–Neotropics using two mitochondrial genes, *COI* and *16S rDNA (16S)*, and a nuclear gene *(ITS2)* in ticks collected at different sites of the Neotropics, in addition to a set of sequences with available coordinates downloaded from GenBank. The main aims of the study are addressed in the following questions: (a) How many clades of the *R. microplus* complex exist in the Neotropics? (b) Is there a spatial gradient of genetic variability among samples in the target region? (c) Does climate impact the divergence of the target genes among sites since it has major restrictive traits for the tick?

## 2. Materials and Methods

### 2.1. Collection of Ticks and Preparation of Samples

**Figure 1:**
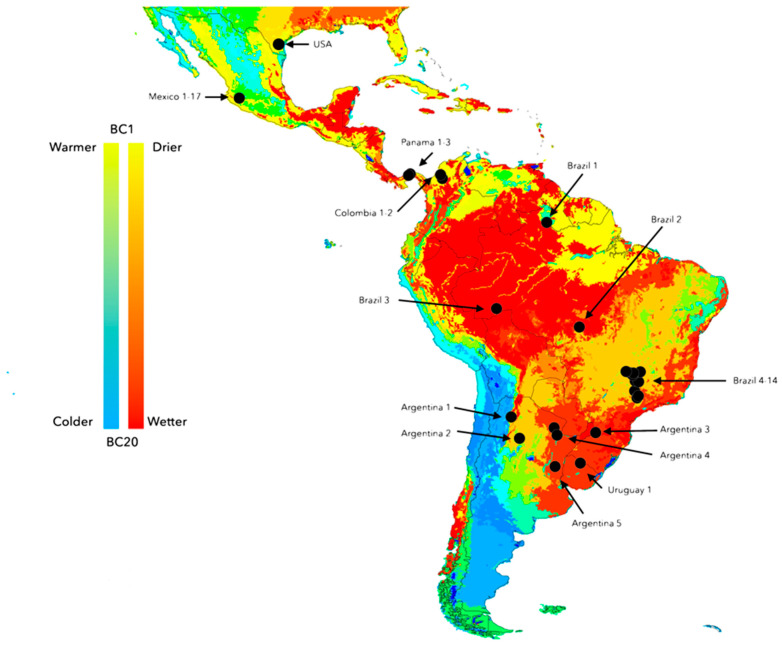
The collection sites of *Rhipicephalus microplus* used in this study in the Nearctic–Neotropical region, overlying the classification of environmental traits in 20 bioclimatic regions (colors). The colors are random and intended only to show the differences and combinations of both temperature and soil humidity. Scales of colors range from the warmer and drier site (arbitrarily named “bioclimatic region 1” or BC1) to the colder and more humid (named “bioclimatic region 20” or BC20). See Material and Methods for the calculation of the bioclimatic regions. Some populations of ticks were collected in near places, separated by no more than 100 km, but subjected to different climate traits. The scale of the map does not allow for the separate plotting of each population, which are included as only one point but labelled accordingly.

All ticks were collected as engorged females at selected points of Central and South America, from Mexico to Argentina (Figure 1). We assigned the term “strain” to each population of ticks collected in different sites, including those selected from GenBank (see below). This term is used to reflect samples collected at sites at variable distances and/or supporting different climate stress. All the ticks were individually identified to species level before further proceeding with the oviposition and sequencing of the eggs. Samples from the Neotropics were either *R. annulatus* or *R. microplus*; each female was stored separately in an incubator. Field-collected engorged females were allowed to oviposit under controlled conditions in incubators (27 °C, 85% relative humidity). Only egg masses coming from a minimum of 10 females were used in this study. The egg masses from the females collected in each site were mixed and processed together. We decided to use the egg masses after identification of each female because they represent the variability of the “strain” in the site of collection and not from only one individual specimen. The workload is significantly reduced without loss of information. All the samples, geographical origin, source, sample IDs, and BOLD/GenBank accession numbers are provided in Supplementary File S1. The collection points were selected based on the prevailing climate features, providing a wide representation of the sites colonized by *R. microplus* for a phylogeographical analysis. Two of the strains (Mexico “La Joya” and Uruguay “Mozo”) are reference strains that animal health authorities keep for studies on resistance of *R. microplus* to acaricides and vaccine efficacy; since these trains are confined to laboratory studies only, no recombinations with other populations should be expected. These strains are also of importance in this context because, having being kept in the laboratory for several years now, they are not subjected to selection by climate stress. We also included in the analysis DNA of strains from India and Pakistan, all of them belonging to the “clade C” of *R. microplus* as previously defined [1,6,7], and DNA from field-collected strains of *R. annulatus* from the Nearctic region (Mexico) to perform a finer phylogenetic comparison.

### 2.2. DNA Extraction, PCR, and Sequencing

Total DNA (mitochondrial DNA and genomic DNA) was extracted using DNeasy Blood and Tissue kit (Qiagen, Hilden, Germany) according to the manufacturer´s instructions. Fragments of two mitochondrial genes, *16S*, *COI*, and the nuclear *ITS2* were amplified via PCR using the primers listed in Table 1. A mounting number of studies hold up the benefits of using more than one DNA genetic marker to assess *Rhipicephalus* phylogenies and increment the phylogenetic resolution at the species level [1,28,29,30]. Amplification of the mitochondrial *COI* was performed at a total volume of 50 µL containing 2 µL of DNA template, 25 µL H_2_O, 5 µL NH_4_ buffer, 5 µL of dNTPs (2 mM/µL), 2.5 µL of MgCl_2_ (25 mM/ul), 0.1 μL Bioline Taq Polymerase (Bioline Reagents Ltd., London, UK), 5 µL of each primer (each at 10 pmol/µL), and 0.38 µL of Bovine Serum Albumin (20 mg/mL). The thermocycler conditions are detailed in Table 1. Additionally, the amplification of the *16S* and *ITS2* genes was performed at a total volume of 25 μL containing 12.5 μL Platinum™ II Hot-Start PCR Master Mix (2×) (Invitrogen), 1 µL of each primer (each at 10 pmol/µL), 10.5 μL of nuclease-free water, and 1 μL genomic DNA. PCR amplification was conducted as shown in Table 1. All PCR products were visualized on a 1.5% agarose gel and samples showing bands of the correct size were bidirectionally sequenced at the Sequencing Unit, Animal and Plant Health Agency (APHA) for the *COI* gene and by SecuGen (Madrid, Spain) for *16S* and *ITS2*.

### 2.3. Alignment and Phylogenetic Analysis

Paired bi-directional sequences were combined to generate a single consensus sequence, which was visually inspected, edited, and trimmed to the same length to remove ambiguous ends using the Geneious Prime v2.2 software (https://www.geneious.com, accessed on 16 June 2021) and was aligned using MAFFT v.7.304 [34]. To obtain a better geographical representation of the haplotypes obtained across the Asian–Nearctic–Neotropic distribution, a first alignment for each marker (*COI*, *16S*, and *ITS2*) was performed including sequences available in GenBank based on taxonomy, the quality of the sequence and their geographic origin (Supplementary File S1). Analysis length was defined as follows: for *COI*, 658 bp alignment in 76 sequences; for *16S* r, 446 bp alignment in 74 sequences; and for *ITS2*, 1379 bp sequence length in a set of 47 sequences. Another alignment was carried out via concatenation of the mitochondrial markers *COI* and *16S* using the Concatenate Sequences tool in Geneious Prime v2.2 software and obtaining 33 sequences of 1017 bp. The phylogenetic relationships of concatenated mitochondrial genes (*COI*+*16S*) were constructed using the haplotypes from the ticks collected and sequences available from GenBank from the Neotropics with both mitochondrial genes available. Lastly, a third alignment for each marker (*COI*, *16S*, and *ITS2*) was performed, using the haplotypes obtained in this study and retrieved from the GenBank from the Nearctic–Neotropics, following the method described above. Analysis length from this alignment was as follows: for *COI*, 608 bp alignment in 44 sequences; for *16S*, 432 bp alignment in 49 sequences; and for *ITS2*, 26 sequences of 1504 bp sequence length. From this third alignment, the genetic distance between each pair of sequences (% Identity) was obtained for each gene in Geneious Prime v2.2 software to perform the analysis described below in Section 4 (Supplementary File S2). Full details for each specimen and sequence information can be found at the Barcode of Life Database (BOLD) (https://www.boldsystems.org, accessed on 16 August 2022) within the “Human Pathogens and Zoonoses Initiative”, Working Group 1.4, BOLD project RHMCP DNA Barcoding world *Rhipicephalus microplus* (eggs).

Phylogenetic relationships of three genes (*COI*, *16S*, *ITS2*) and concatenated mitochondrial genes (*COI*+*16S*) were analyzed using maximum likelihood (ML) and Bayesian inference (BI) frameworks. ML was performed using MEGAX software [35] after selecting the best-fit substitution model in jModelTest2 v.2.1.10 based on the Akaike Information Criterion (AIC) [36]. The support of the resulting nodes was estimated using 1000 bootstrap replicates in MEGAX. BI was performed using Mr. Bayes version 3.1.2 [37,38] using the best-fitting mutation model for each gene as mentioned above. Markov Chain Monte Carlo (MCMC) chains run for 1,000,000 generations, sampling every 200 generations, with the first 1000 sample trees discarded. Convergence of split frequencies was considered when an average standard deviation reached values below 0.01. Phylogenetic tree annotation and visualization were performed using FigTree v1.4.4 (http://tree.bio.ed.ac.uk/ accessed on 16 May 2021) [39]. 

### 2.4. Haplotype Networks and Genetic Diversity of R. microplus

Genetic diversity parameters were assessed for each gene marker (*COI*, *16S*, *ITS2*) and concatenated mitochondrial markers (*COI*+*16S*) grouping haplotypes within the *R. microplus* complex by geographical region using DnaSP v6 software [40]. The number of haplotypes, haplotype diversity (Hd), nucleotide diversity (pi), number of segregating sites, and alignment size (bp) were included (Supplementary File S3). To visualize the genetic relationships of the haplotypes studied within the *R. microplus* complex, we performed a haplotype network for each marker gene, using the TCS statistical parsimony algorithm in the POPART software package [41]. 

### 2.5. Climate and Genetic Variability among Populations of Neotropical R. microplus

Once the phylogenies were built for each gene (*COI*, *16S*, *ITS2*), we aimed to test the correlation of the phylogenies in the Nearctic–Neotropical samples of *R. microplus* with selected environmental features known to impact the life cycle of the ticks, including the temperature (Land Surface Temperature, LST), which regulates the development of the molting stages of the tick), and the vegetation stress (Normalized Derived Vegetation Index, NDVI, a proxy for relative humidity, which regulates tick mortality). The focus is to investigate if environmental features could “filter” the species, promoting adaptations to local combinations of LST and NDVI, detectable in the target genes. We only used the geo-referenced records of the Neotropics for this part of the study because each strain of the tick must be ascribed to the environmental values. To note, the hypothesis is not that climate could impact specifically the target genes selected because they have a proven capacity to resolve the different clades of the ticks [1] and are widely available in GenBank, providing enough samples for comparison.

Environmental data were obtained from the MODIS satellite repository (https://modis.gsfc.nasa.gov, last accessed June 2022) at monthly chunks and a spatial resolution of 0.05°; they were converted to the monthly averaged values for the complete period 2001–2020. This resulted in two sets of variables covering the 12 months of the averaged years. We approached a classification of the target region based on a reduction of the environmental values (LST and NDVI) using a Principal Components Analysis. We classified the territory in “clusters” of statistically similar LST and NDVI values. Stacks with the 12 monthly layers of the two variables mentioned above, averaged as explained, were used for an unsupervised classification of the territory (total: 24 variables) using Principal Components Analysis. The purpose is to classify and group portions of the territory on the grounds of LSDT and NDVI. We adhered to the k-means algorithm incorporated in the software Erdas Imagine (Hexagon Software) to process the set of raster maps resulting in the bioregionalization based on the two variables. To implement a *k*-means classification algorithm, a target number of regions *k* was determined by maximizing the cluster validity index. The Calinski–Harabasz Variance Ratio Criterion [42] was used to measure within-group and between-group dispersion. The classification produced a set of areas representing a unique combination of environmental values, as summarized in Figure 1. This is one of the reasons for the use of populations of ticks (i.e., specimens collected in the same biogeographical area) instead of specimens, because the size of the biogeographical area is large, and all specimens collected “inside” such an area will receive the same biogeographical background. No biogeographical differences that could be applied between specimens would be observed, and therefore, even if individually sequenced, the possible differences would remain unnoticed.

Clusters belonging to different categories are statistically different from others within the margins of the k-means algorithm; they have also a measurable environmental distance among them regarding the differences of the climate variables. We calculated the environmental distance among categories via the Schoener’s D distance using the package ENMTools [43] for R [44] which calculates a Euclidean distance based on the values of monthly LST and NDVI among clusters. This was another reason to work on populations of ticks instead of individuals, because then the number of distances among specimens would increase largely, further complicating the interpretation of the results. On the other side, there are two important tests for associating the genetic distances among strains and the environmental values, namely Blomberg’s K and the Pagel’s lambda. They both measure a phylogenetic signal and compare the observed signal in a trait to the signal under a Brownian motion model of trait evolution on a phylogeny [45,46]. Here, we are interested in demonstrating the phylogenetic signal behind the environmental distances. K values of 1 correspond to a Brownian motion process. K values closer to zero correspond to a random pattern of evolution, while K values greater than 1 indicate strong phylogenetic signal and conservatism of environmental features. Pagel’s lambda scales between 0 and 1. Values of 1 mean a high phylogenetic signal and strong correlation with the variation in the environmental variable. In evolutionary terms, this refers to tick lineages fitting divergent conditions represented by the clusters obtained from climate variables. The alternative (lambda = 0) is that climate variables vary differently with respect to a Brownian motion model and do not correlate with a tree; this is indicative of a low phylogenetic signal, in which populations do not distinctly fit climate patterns proportional to the tree branch lengths. This could be due to total randomness in genetic drift. We obtained the phylogenetic signal in the matrix of environmental distances using the package *phytools* [47] for R.

### 2.6. Geographic Distance among Populations as Possible Driver of Genetic Variability of R. microplus

Other than the possible molecular divergence of the target genes according to environmental features, phylogenetic dissimilarity among Neotropical populations could occur because a large geographic separation and consequently a lack of exchange of specimens among sites may prevent interbreeding. The phylogenetic signal was used as before to calculate both Pagel’s lambda and Blomberg’s K to verify the correlation between the genetic distance among the strains of the Neotropical *R. microplus* and the geographic distance among samples. Geographic distance matrices were generated using the latitude and longitude points of each collection point with the package *geodist* [48] for R. The *multiPhylosignal* function was used to compare the genetic similarity among strains and the logarithm (base 10) of the distance in kilometers. To limit inconsistences in the results, we used logarithmic transformation of the data to adjust large variability in the distance among strains; i.e., some strains may be separated by a few hundred km (i.e., Uruguay and Argentina), while others may be separated by thousands of km (i.e., Mexico and Argentina).

## 3. Results

### 3.1. Only R. microplus s.s. (Clade A) Exists in the Nearctic–Neotropics

**Figure 2:**
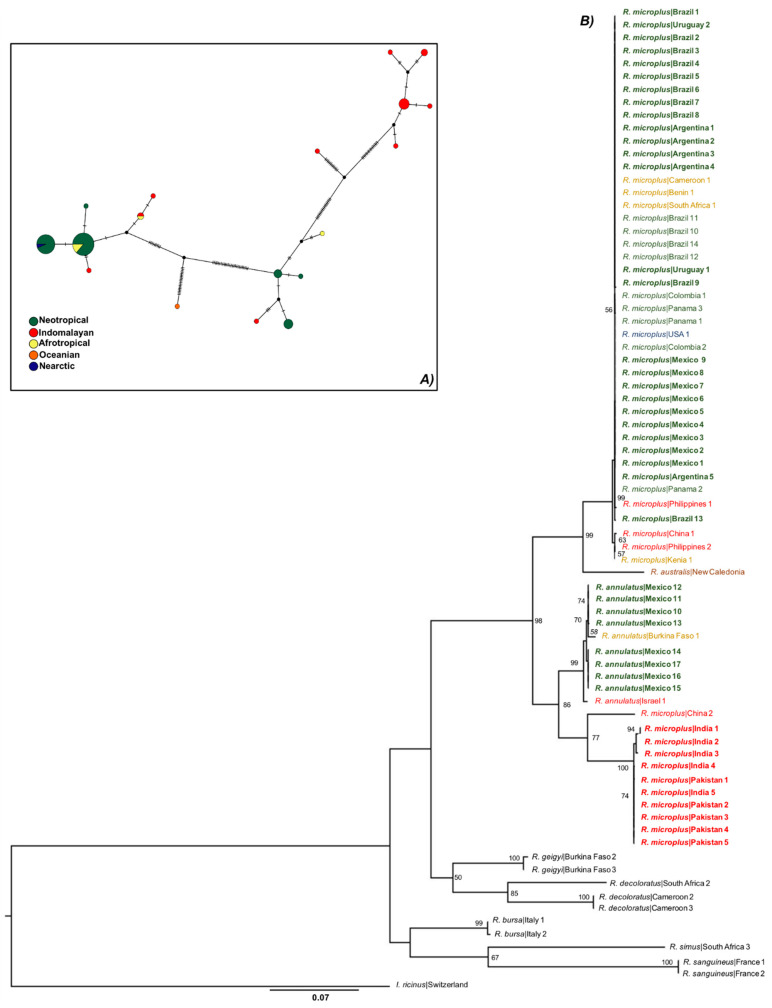
(**A**) Haplotype network for the *COI* gene. Major circles represent predominant haplotypes. A branch represents a single nucleotide change and lines on branches represent inferred missing haplotypes. Colors of the nodes show the locations where haplotypes were collected. (**B**) Asian–Neotropical maximum likelihood tree inferred with partial sequences of partial *COI* mtDNA (*COI*) using the GTR+G+I model of nucleotide substitution. Sequence data generated in the present study are highlighted in bold. Retrieved sequences from GenBank with the accession numbers and geographical origin are available in the Supplementary File S1 and were included to generate a robust phylogenetic tree. Support values were indicated at each node (bootstrap < 50% are not shown). The bar represents 0.07 substitutions per site. The tree was rooted using Ixodes ricinus as outgroup.

**Figure 3:**
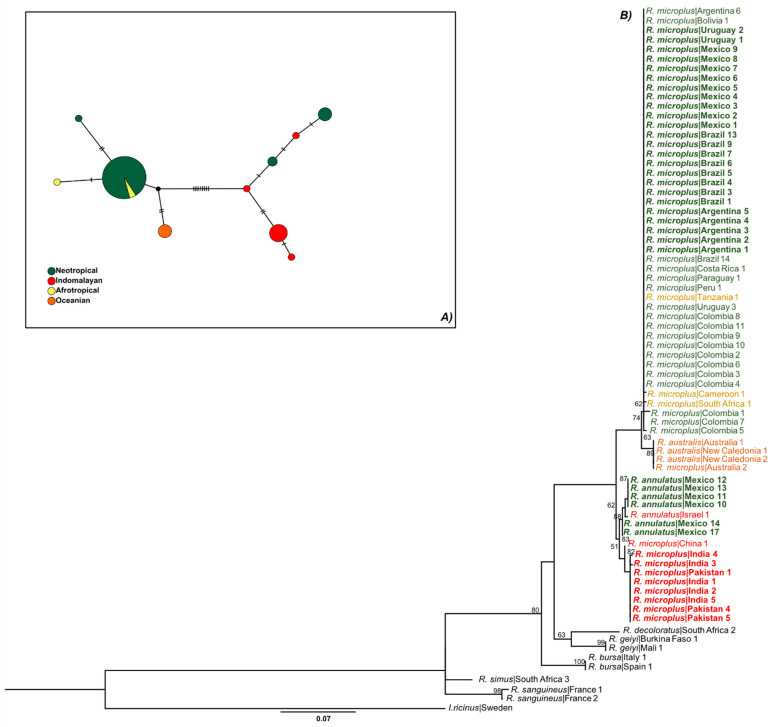
(**A**) Haplotype network for the *16S* rDNA gene. Major circles represent predominant haplotypes. A branch represents a single nucleotide change and lines on branches represent inferred missing haplotypes. Colors of the nodes show the locations where haplotypes were collected. (**B**) Asian–Neotropical maximum likelihood tree inferred with partial sequences of the *16S* rDNA (*16S*) using the GTR+G model of nucleotide substitution. Haplotypes generated in the present study are highlighted in bold. Retrieved sequences from GenBank with the accession numbers and geographical origin are available in Supplementary File S1 and were included to generate a robust phylogenetic tree. Support values are indicated at each node (bootstrap < 50% are not shown). The bar represents 0.07 substitutions per site. The tree was rooted using Ixodes ricinus as outgroup.

**Figure 4:**
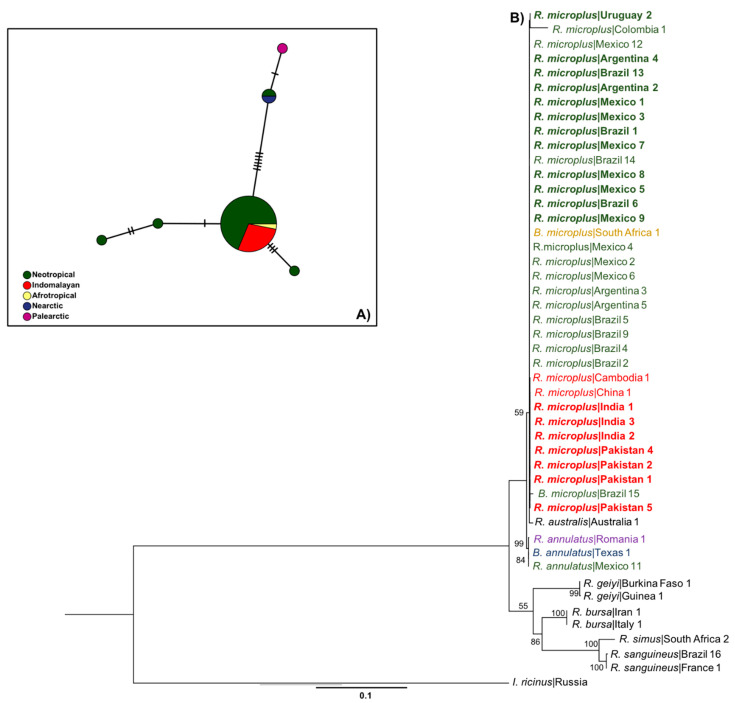
(**A**) Haplotype network for the ITS2 gene. Major circles represent predominant haplotypes. A branch represents a single nucleotide change and lines on branches represent inferred missing haplotypes. Colors of the nodes show the biogeographical locations where haplotypes were collected. (**B**) Asian–Neotropical maximum likelihood tree inferred with partial sequences of the Internal transcribed spacer (ITS2) sequences using the GTR+G model of nucleotide substitution. Haplotypes generated in the present study are highlighted in bold. Retrieved sequences from GenBank with the accession numbers and geographical origin are available in Supplementary File S1 and were included to generate a robust phylogenetic tree. Support values are indicated at each node (bootstrap <50% are not shown). The bar represents 0.1 substitutions per site. The tree was rooted using Ixodes ricinus as outgroup.

**Figure 5:**
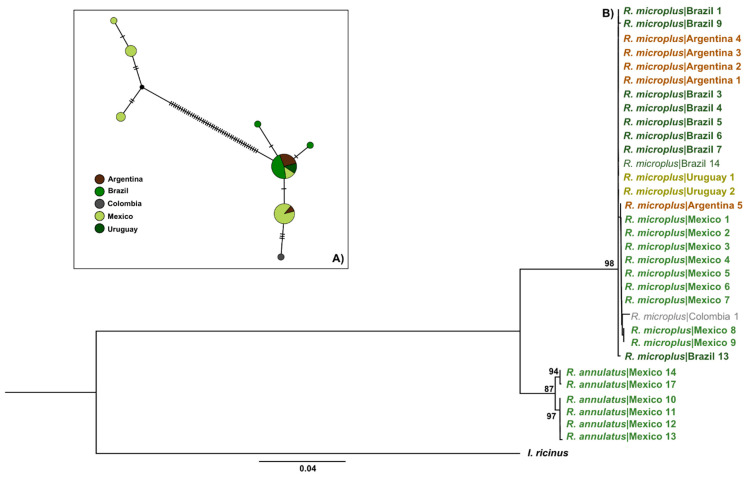
(**A**) Nearctic–Neotropic maximum likelihood tree inferred with concatenated partial sequences of the mitochondrial genes *COI* and *16S* rDNA using the GTR+G model of nucleotide substitution. Haplotypes generated in the present study are included in (**B**); support values are indicated at each node (bootstrap < 50% are not shown). The bar represents 0.04 substitutions per site. The tree was rooted using Ixodes ricinus as outgroup.

According to the haplotype network (see below), the *COI* mitochondrial marker showed a total of 18 haplotypes, three of which were only present in the Neotropical regions, separated by several sequence mutations from lineages detected in other areas (Figure 2A). Meanwhile, 9 different haplotypes were exclusively from the Indomalayan region. Interestingly, the Indomalayan, Afrotropical, and Nearctic populations shared haplotypes with the Neotropical population (Figure 2A). A total of 10 haplotypes were observed from the analysis of *16S*, three of them were observed only in the Neotropical populations, four of them in the Indomalayan, and only one from the Afrotropical and Oceanian biogeographical regions (Figure 3A). We only observed shared haplotypes of *16S* between the Neotropical and Afrotropical populations. For the ITS2 marker, the haplotype network revealed six different haplotypes, one of them shared by the Neotropical, Indomalayan, and Afrotropical populations (Figure 4A). Focusing on the haplotype network from the concatenated mitochondrial markers of the Neotropic haplotypes, we observed a total of eight haplotypes (Figure 5A), three of which were only present in Mexico, and separated by several sequence mutations from other haplotypes belonging to this region and other countries. Also, two of the haplotypes were exclusively detected in Brazil, and one in Colombia.

The ML (Figure 2B, Figure 3B and Figure 4B) and BI (Supplementary File S4: Figures S1–S3) phylogenies of *R. microplus* haplotypes of mitochondrial genes (*COI*, *16S*) showed similar topologies. The mitochondrial gene *COI* (Figure 2B) showed strong support (99%) for the clade that includes all the Nearctic–Neotropical *R. microplus*, and moderate support for the clade that included the *R. microplus* strains from China, the Philippines, and Kenya (63%). The *R. microplus* clade is closely related to the *R. annulatus* strains (98%) which are also sisters of *R. microplus* from India, Pakistan, and China (86%). Thus, the *COI* ML tree successfully resolved the relationships within the *R. microplus* complex and supported monophyly for *R. annulatus* and *R. microplus* in Neotropics (Figure 2B). Meanwhile, the *16S* ML tree (Figure 3B) showed weak support for the *R. microplus* cluster and for its division in the clades that included *R. microplus* from Nearctic–Neotropics and African strains, plus *R. microplus* from Asian strains (less than 50% bootstrap, not shown in the phylogenetic tree). However, there is good support among these clades for the monophyly of *R. annulatus* from Mexico (87%) and Israel, *R. microplus* from India (82%), and R. australis from Australia and New Caledonia (89%). A similar result was observed on the concatenated *COI*+*16S* ML phylogeny, showing all haplotypes of *R. microplus* from the Neotropics grouped under the same clade (98%) and supporting monophyly for the *R. annulatus* clade (97%) (Figure 5B). The ITS2 ML tree (Figure 4B) showed weak support for the *R. microplus* clade (59%), and thus poorly reflects the relationships within such a complex. The nuclear marker was unable to differentiate between the Nearctic–Neotropics and the Asian clades of *R. microplus*, whereas it correctly identified the *R. annulatus* (99%), *R. bursa* (100%), *R. sanguineus* s.l. (100%), and *R. geigyi* clades (99%).

**Figure 6:**
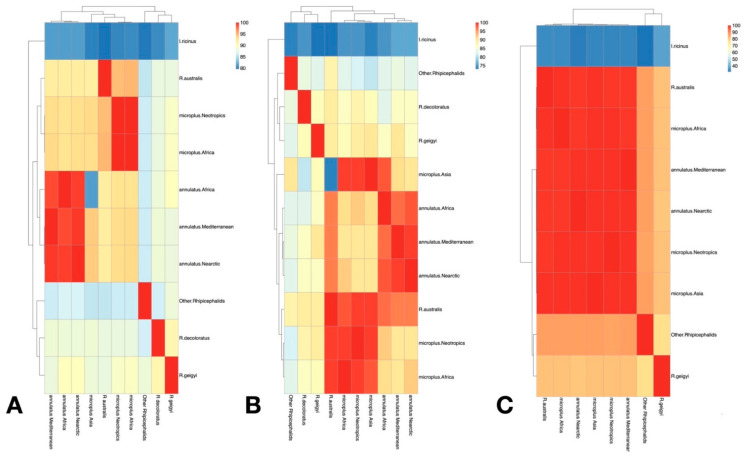
Heat maps displaying the percent similarity in the *COI* (**A**), *16S* rDNA (**B**), and ITS2 (**C**) sequences for groups of species and collection areas included in this study. A dendrogram (left of each chart) is provided to show the averaged genetic similarities of each gene among samples.

We plotted the mean percent of identity among the different haplotypes using the sets of *R. microplus* (Neotropical, African, Asian) and *R. annulatus* (Nearctic, African, Mediterranean), testing separately against *R. australis*, *R. decoloratus*, *R. geigyi*, other *Rhipicephalus* spp., and the outgroup *I. ricinus* (Figure 6). The results demonstrated the high similarity of the *R. microplus* collected in Africa and the Neotropics with the samples of the clade A. Asian representatives of clade C are well separated from the samples above. Results strongly support that only *R. microplus* clade A is present in the Nearctic and Neotropics. In the same way, *R. annulatus* collected in places of the Nearctic, Africa and the Mediterranean region are almost identical. This high similarity of Neotropical and African populations of *R. microplus* and *R. annulatus* supports the utility of *COI* and *16S* for tracking the status of populations that became separated probably hundreds of years ago, even if both populations (African and Neotropical, of either *R. annulatus* or *R. microplus*) came from different invasive events at different moments of the timeline. Supporting previous results, ITS2 was unable to separate the main groups of Boophilus; only species belonging to the main outgroup and to “other rhipicephalids” were adequately separated with this nuclear marker.

### 3.2. Climate Traits Are Not Driving the Mutation Rates of Three Genes of R. microplus

We argued that the gradient of climate in the Neotropics could be a driver of adaptation of *R. microplus* to the local conditions of the environment. The hypothesis is that most similar populations should colonize regions with similar climate patterns; the opposite option is that the tick has a large phenotypic plasticity to adapt to a range of conditions and that such phylogenetic signature of the environmental traits may be present in the target genes. A Bloomberg’s K test comparing differences of climate and genetic similarities among strains showed that there are no relationships between climate patterns and the phylogenies obtained for *COI*, *16S*, and ITS2 of the geo-referenced populations of *R. microplus* (test value = 0.04, *p* < 0.01; test value = 0.03, *p* < 0.03; test value = 0.01, *p* < 0.01, respectively, for each gene). The test value near zero and the high *p*-value indicated that the variability is almost random and that climate patterns detected from remotely sensed information do not affect the mutation rates of *COI*, *16S*, or ITS2. The Lambda test also failed to find a correlation among climate and genetic similarities among strains (test value = 0.12, *p* = 0.019; test value = 0.18, *p* = 0.03; test value = 0.06, *p* < 0.01, respectively, for *COI*, *16S*, or ITS2).

### 3.3. There Is a Random Pattern of Genetic Variability of R. microplus According to Distance in Neotropics

We tested whether spatial distance influences the genetic variability of the three genes included in this study. The most feasible hypothesis is that more distant individuals should be less related, because widely separated strains lack exchange of specimens (to note, the hypothesis of introduction of ticks while feeding cannot be tested for such large territory). The Bloomberg’s K test of similarity comparing genetic variability and geographic distance produced values of −0.44 (*p* < 0.01) for *COI*, −0.21 (*p* < 0.05) for *16S*, and −0.05 (*p* = 0.633) for ITS2. Lambda’s test of similarity for the same features produced test values of 0.12 (*p* < 0.01) for *COI*, 0.05 (*p* < 0.05) for *16S*, and 0.02 (*p* = 0.633) for ITS2. These results are indicative of a random genetic evolution; in other words, far populations are not more (dis)similar than close ones. Therefore, we hypothesize that geographical distance does not impact the small genetic differentiation between the populations of *R. microplus* in the Neotropics. The hypothesis that closer populations of *R. microplus* in the Neotropics should be more similar than those geographically separated is rejected by our results.

## 4. Discussion

This study provided new insights into the current phylogenetic structure of the *R. microplus* complex in the Neotropics. For that, the ML and BI phylogenies of *R. microplus* were constructed based on two mitochondrial markers, *COI* and *16S*, and the nuclear marker *ITS2*, as previously described in similar studies [1,26,28,30,49,50,51,52]. We further compared data obtained from field collections with sequences from different biogeographical regions available in GenBank to provide additional support for the phylogenetic relationships of the tick subgenus *Boophilus*.

Overall, the *COI* DNA barcoding analysis provided higher resolution. The *R. microplus* complex showed five major clades. The phylogenetic tree based on *COI* clearly differentiated *R. annulatus* from the other clades of *R. microplus* with strong support (77–100%). A similar topology using *COI* was observed in India [10], showing a clear differentiation of the *R. microplus* clades from the *R. annulatus* in haplotypes. In this study, we demonstrated that haplotypes of *R. microplus* collected (or with available sequence(s)) in the Neotropical region belong to clade A of the complex of species *sensu* [1,6,7]. This is an important finding because, after the reclassification of these cryptic species into clades, it was important to test the probable existence of different clades in the Nearctic–Neotropics. With less node support, the phylogeny based on *16S* also revealed four well-differentiated clades that differentiate *R. microplus* clade A from *R. australis*, *R. annulatus*, and a group of *R. microplus* haplotypes that belong to the clade C as previously defined [7]. The phylogenetic relationships among the few samples of the Asian *R. microplus*-like ones that were included in the study did not show differences to samples from Indian and Pakistani strains, grouping together in a tight cluster. Only one sample from China splits from that cluster. These results agree with previous findings describing the group of cryptic species existing in the Asian region [1]. These clades are not yet formally defined at a specific level and referred to as “*R. microplus*” because of the lack of tests on crosses and back-crosses under controlled conditions among representatives allowing species delimitations (a fertile progeny versus a hybrid one). Morphological studies exist for specimens of the so-called Clade C [1] as well as for *R. australis* [53].

Focusing on the genetic data of the *R. microplus* complex in the Neotropics, the phylogeny constructed after concatenation of *COI* and *16S* showed a strong differentiation of the *R. microplus* clade A, and the *R. annulatus* from Mexico. The phylogenetic tree constructed using the *ITS2* was not able to resolve the phylogenetic relationships within the *R. microplus* complex, *R. microplus* clade A and C, and *R. australis*, but it was able to differentiate a clade for *R. annulatus*. This result was previously reported by other authors [1,10], showing the limitations of using the *ITS2* for differentiation of related species within the *R. microplus* complex. Other studies demonstrated that the nuclear marker *ITS2* resulted in poor resolution for the phylogeny of the cryptic species of *R. microplus* in comparison with mitochondrial genes [6,10].

According to the haplotype network, the *COI* mitochondrial marker demonstrated how Indomalayan, Afrotropical, and Nearctic populations shared haplotypes with Neotropical populations. Shared haplotypes between the Neotropical and Afrotropical populations were also observed for *16S*. For the *ITS2* marker, the haplotype network revealed six different haplotypes, one of them shared by the Neotropical, Indomalayan, and Afrotropical populations. These findings support the hypothesis of one or few invasive events of *R. microplus* clade A in the southern Nearctic–Neotropical area. Although it is yet unknown how and when the species was introduced, a radiation from Indomalayan and Afrotropical biogeographical regions can be suggested, with further and very recent importations into Africa. 

When assessing phylogenies with mitochondrial *COI* and *16S* haplotypes of *R. microplus*, we observed a low variability and genetic uniformity in the Nearctic–Neotropical region. However, both genes are enough to separate the different clades of the *R. microplus* complex. Accordingly, *COI* was chosen as the only marker for providing evidence of *R. microplus* in Kenya and Sub-Saharan Africa [54] and India [10], for studying the taxonomic status of the complex of species [55], or for checking the population variability of the tick in South Africa [8]. The finding of a lack of correlation between the genetic distances of the *COI* and *16S* genes among the geo-referenced samples of *R. microplus* and the geographic distance confirms that populations tend to be genetically similar at long distances. Crosses of *R. microplus* over long distances seem to be common, perhaps derived from cattle trade over long distances, which highlights inadequate tick control in some cases [54]. *R. microplus* is distributed along a continuum over the continent, excluding patches where the climate is locally unsuitable, adequate hosts are absent or where active control campaigns persist. Under these conditions, we hypothesize that the contact among cattle herds kept in contiguous ranches is responsible for the short-distance inter-breeding of ticks, while uncontrolled movements of livestock may be responsible for long-distance mixing. The hypothesis of the use of wild ungulates as hosts for long-distance travel allowing mixing of distant populations should not be rejected, since the tick feeds for 24 days. This has been also discussed [10] regarding the lack of a genetic signature of tick populations because of long-distance movements of cattle. These results, however, are not compatible with the data by [56] using microsatellite markers of *R. microplus* in Texas, who reported a genetic structure of microsatellites in a pattern of isolation-by-distance on the short scale.

Another factor that could shape the results obtained for *R. microplus* is the short time the tick has been spreading into the region. The approximate date and place of introduction are unknown, but the Neotropical populations are not yet significantly separated from others belonging to the clade A in Asia, the presumed ancestral area of this species. Arguments for *R. annulatus* support this hypothesis. *Rhipicephalus annulatus* has a disjoint distribution in the Mediterranean and Sub-Saharan Africa [57]. The formation of the Sahara Desert, that most probably split both northern and southern populations of *R. annulatus*, is still a matter of debate [58] but it is measured in a minimum of thousands of years. Considering that both *COI* and *16S* of the Tropical and Mediterranean populations of *R. annulatus* are very similar, and that this similarity persists in Nearctic samples (spread only about 300–400 years ago [59]), a conclusion could be that the mutation rates of both genes is low enough to measure the divergence at these timescales. Results point to a few introductions (or only one event) of *R. microplus* in the Neotropics spreading throughout suitable environments and available hosts in the surveyed region, and without yet enough clues to evaluate the evolution of the strains.

Since published data suggest a high ability of *R. microplus* to adapt to a large range of climate features in sites where it has been introduced, we expected a measurable genetic variability correlation between the phylogenies and some climate features. However, results did not support this hypothesis as the small changes in examined sequences remain uncorrelated with these features. For example, strains of *R. microplus* kept under laboratory conditions at constant conditions of temperature, humidity, and light regimes, commonly used for analyses of sensitivity to acaricides and vaccination trials, have a similar sequence to wild strains obtained from field surveys. It appears that field populations subjected to changes in the climate patterns did not show major differences in the sequences of the three genes, compared with those of laboratory colonies that are kept under constant environmental conditions. This is an additional clue supporting the idea that climate is not the driver of the small variability of the tested genes. It is necessary to consider the special features of the life cycle of the target species as the only tick subgenus that has a one-host life cycle. This means that the three stages of the tick’s life cycle feed consecutively on the same host, avoiding searching for hosts three different times, and reducing mortality produced by climate stress. This fact could be behind the lack of “environmental signature” in the samples sequenced, a consistent result supported by two different statistics applied to samples in the whole Nearctic–Neotropical region.

It remains to be investigated whether the adequate markers to track these changes were used, or if other genes could be better for measuring the impact by these traits on the tick populations, but we did not manage to select adequate markers for these changes. As supported by previous data, the nuclear *ITS2* produced poorer results when used on *Boophilus* [6], and mitochondrial genes seem to work better. We encourage the finding and testing of other markers that could help in establishing a relationship between the climate and genetic mutations. Perhaps the sequencing of the complete mitogenome would provide a better overview of these hypothetical associations between genetic structure and environmental traits [60]. Future studies analyzing multiple genome cluster sequences may provide a more comprehensive analysis of genetic variability in ticks. Additionally, gene expression and protein representation analyses may provide additional information on the possible functional implications of genetic variability in species of ticks subjected to a range of features of climate.

## 5. Conclusions

The Nearctic–Neotropical samples of *R. microplus* s.s. belong only to clade A, and they show small differences in the sequences of three target genes even if collected in sites experiencing quite different annual weather patterns. Additionally, the *COI* gene is a very reliable marker of tick species and clade, and therefore its use is supported as one of the methods of the panoply of tools available for genetic studies of ticks. The *COI* and *16S* patterns of *R. microplus* in the Neotropics are consistent with the spread of a monophyletic lineage and a panmixia of populations, probably because of livestock trade and movements of wild ungulates. The structure of the Neotropical populations of *R. microplus* is thus very stable. This genetic structure supports the hypothesis that the interventions based on tick antigens for the control of *R. microplus* cattle infestations may be effective in broad areas of the Neotropics.

Limitations of the study on the use of tick pooled samples, although with limited implications for reported results, should be addressed in future studies by comparing data analysis in pools and individual ticks.

## Figures and Tables

**Table 1:** Genes, primers, and PCR conditions used for amplification of their respective sequences.

Target (Gene)	Primers	Sequence (5′→3′)	PCR Program	Reference Primers
*COI*	LCO1490	GGTCAACAAATCATAAAGATATTGG	Initial denaturation 94 °C, 1 min Pre-amplification: 5 cycles Denature 94 °C, 1 min Anneal 45 °C, 1.5 min Extend 72 °C, 1.5 min Amplification: 35 cycles Denature 94 °C, 1 min Anneal 57 °C, 1.5 min Extend 72 °C, 1 min Final elongation 72 °C, 1 min	[31]
	HC02198	TAAACTTCAGGGTGACCAAAA AATCA		
*16S*	16 + 1	CTGCTCAATGATTTTTTAAATTGCTGTGG	Initial denaturation: 94 °C, 4 min Amplification: 30 cycles Denature 94 °C, 1 min Anneal 54 °C, 30 s Extend 72 °C, 30 s Final elongation: 72 °C, 10 min	[32]
	16 – 1	CCGGTCTGAACTCAGATCAAGT		
*ITS2*	3SA	CTAAGC GGTGGATCACTCGG	Initial denaturation: 94 °C, 4 min Amplification: 30 cycles Denature 94 °C, 1 min Anneal 55 °C, 30 s Extend 72 °C, 30 s Final elongation: 72 °C, 10 min	[33]
	JB9A	GCACTATCAAGCAACACGACTC		

## Data Availability

Complete new sequences as well as Genbank sequences previously deposited and used in this study are included in the Supplementary Materials, together with the references regarding original publications. All the phylogenetic trees generated in this study are available, separated from the main body of the paper, in the Supplementary Materials.

## Supplementary Materials

The following supporting information can be downloaded at: https://www.mdpi.com/article/10.3390/pathogens12111344/s1, Figure S1: Asian–Neotropical Bayesian tree inferred with partial sequences of partial *COI* mtDNA *(COI)* using the GTR+G+I model of nucleotide substitution. Sequence data generated in the present study are highlighted in bold; Figure S2: Asian–Neotropical Bayesian tree inferred with partial sequences of partial *16S* rDNA (*16S*) using the GTR+G model of nucleotide substitution. Sequence data generated in the present study are highlighted in bold; Figure S3: Asian–Neotropical Bayesian tree inferred with partial sequences of partial ITS2 using the GTR+G model of nucleotide substitution. Sequence data generated in the present study are highlighted in bold; Figure S4: Asian–Neotropical Bayesian tree inferred with concatenated partial sequences of the mitochondrial genes, *COI* and *16S* rDNA using the GTR+G model of nucleotide substitution. Tables S1–S3: An excel-type spreadsheet including all the samples and the maximum likelihood reconstruction of the phylogenies based on *COI*, *16S*, or *ITS2* (in separate sheets). Each sheet includes the name as in the figures (phylogenetic trees) as well as the precedence (country), number of GenBank or BOLD accession number and reference (in case it was not from our own collections): Tables S4–S7: An excel-type spreadsheet including the similarities among samples based on *COI*, *16S*, or *ITS2* (in separate sheets). A fourth sheet includes the distance in km among geo-referenced samples (in a straight line). The fifth sheet includes the percent of environmental similarity among the ecological regions outlined for the phylogeographic study. All the data about similarities are in the range 0–100; Table S8: Alignment size (bp), number of segregating sites, number of haplotypes, haplotype diversity (Hd), nucleotide diversity (pi), and number of sequences of *R. microplus*.
