# Supplementary material for: Low Genetic Diversity of the Only Clade of the Tick Rhipicephalus microplus in the Neotropics
Source: Pathogens. 2023 Nov 13;12(11):1344. doi: 10.3390/pathogens12111344 (PMC10675012; doi:10.3390/pathogens12111344)
Supplement: Supplementary file 1 [file pathogens-12-01344-s001.zip › Supplementary File 3.pdf]

Table S8. Number of sequences, number of segregating sites, number of haplotypes (h), haplotype diversity (Hd), and nucleotide diversity (pi), of *R. microplus* complex populations.

Gene/Length (bp)	Region	N sequences	S	h	Hd	pi
**COI/658**	Indomalayan	15	67	10	0.8952	0.0410
	Neotropical	43	40	6	0.6844	0.0223
	Afrotropical	5	41	3	0.7000	0.0317
**16S/446**	Indomalayan	10	5	4	0.5333	0.0056
	Neotropical	48	14	4	0.2668	0.0116
	Afrotropical	3	1	2	0.6667	0.0029
	Oceanian	4	0	1	0	0
**ITS/1379**	Indomalayan	9	0	1	0	0
	Neotropical	26	18	6	0.3539	0.0022
**COI+16S/1017**	Mexico	15	53	4	0.6191	0.0265
	Argentina	5	1	2	0.4000	0.0004
	Brazil	9	2	3	0.4167	0.0004
	Uruguay	2	0	1	0	0
