# Supplementary material for: Low Genetic Diversity of the Only Clade of the Tick Rhipicephalus microplus in the Neotropics
Source: Pathogens. 2023 Nov 13;12(11):1344. doi: 10.3390/pathogens12111344 (PMC10675012; doi:10.3390/pathogens12111344)
Supplement: Supplementary file 1 [file pathogens-12-01344-s001.zip › Supplementary File 4.pdf]

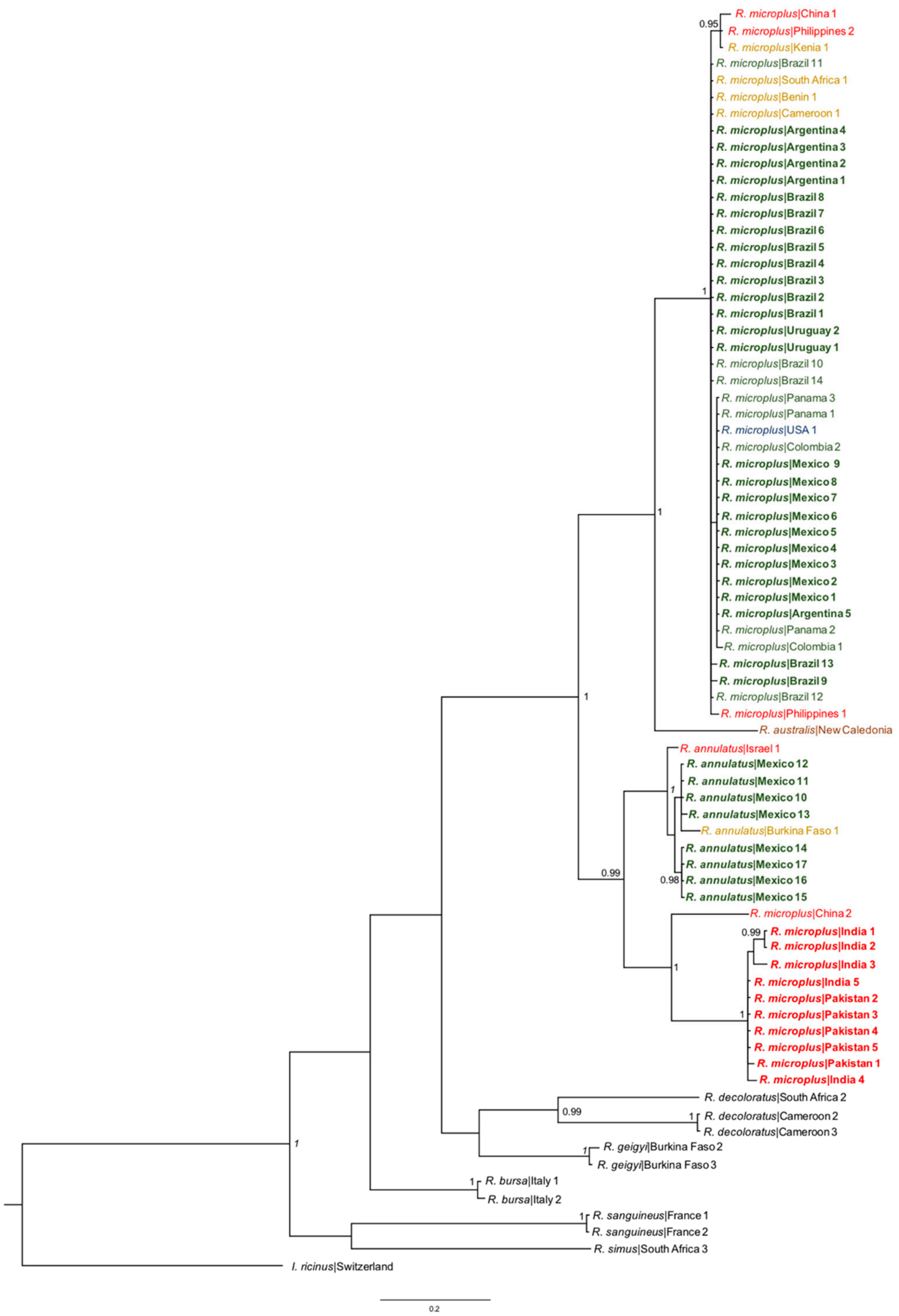

Supplementary File 4. Figure S1.

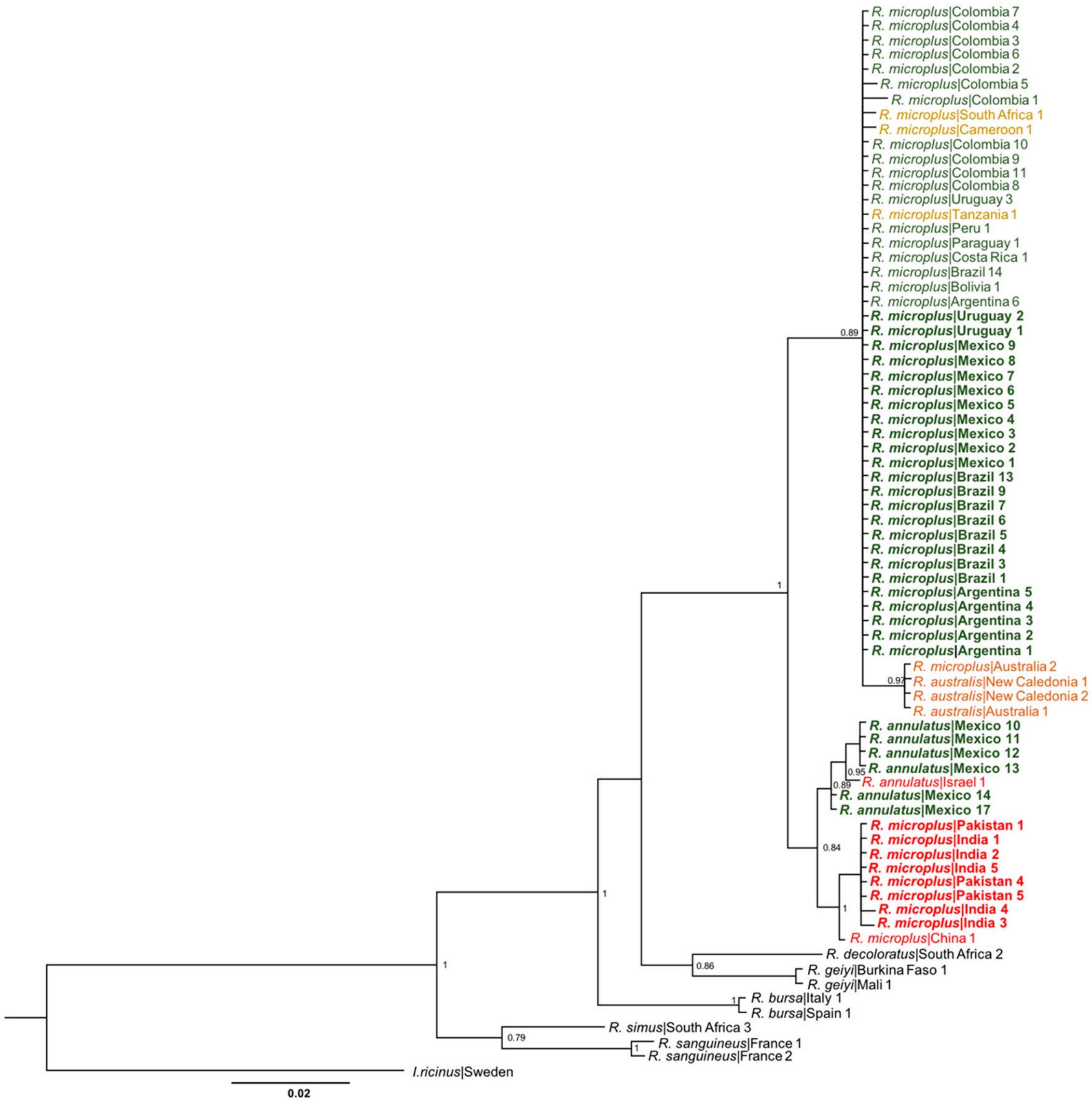

Supplementary File 4. Figure S2.

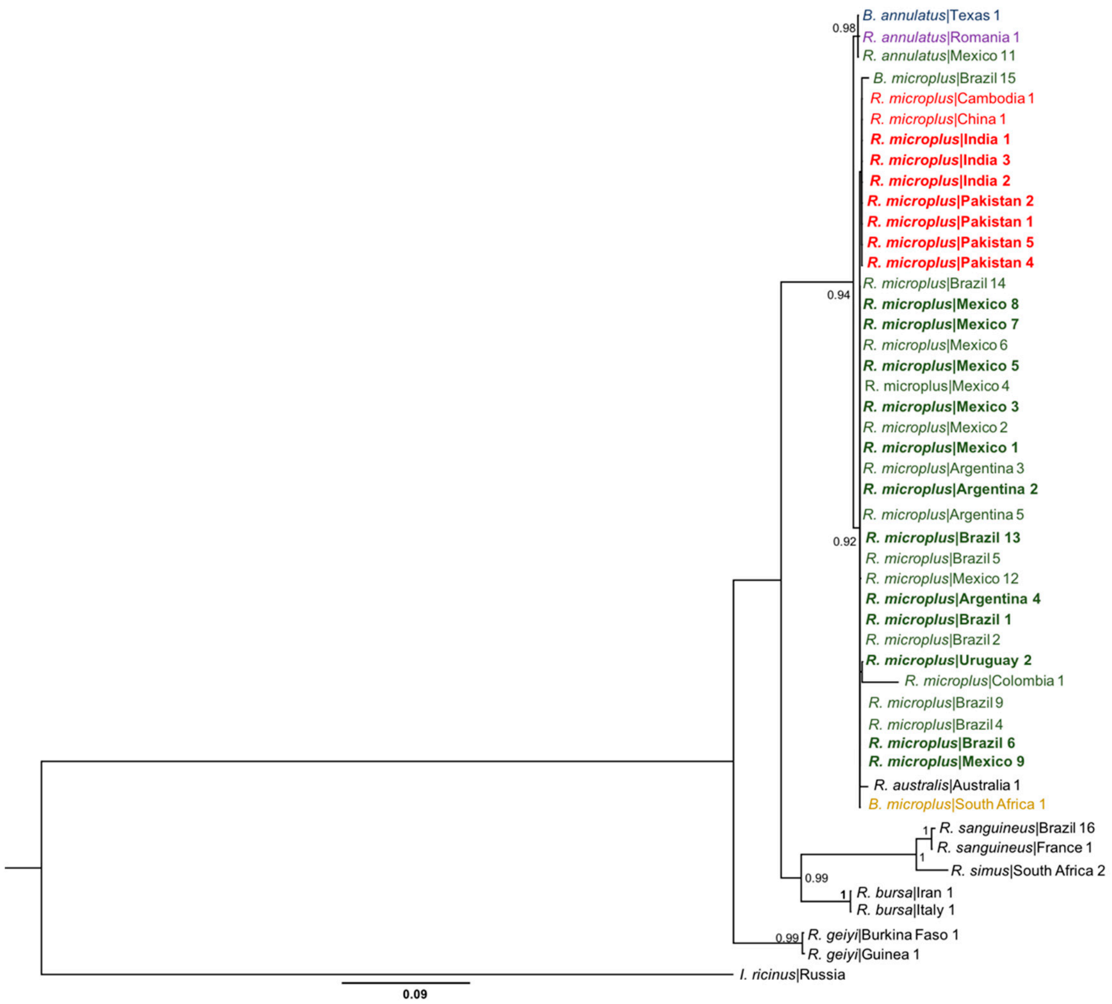

Supplementary File 4. Figure S3.

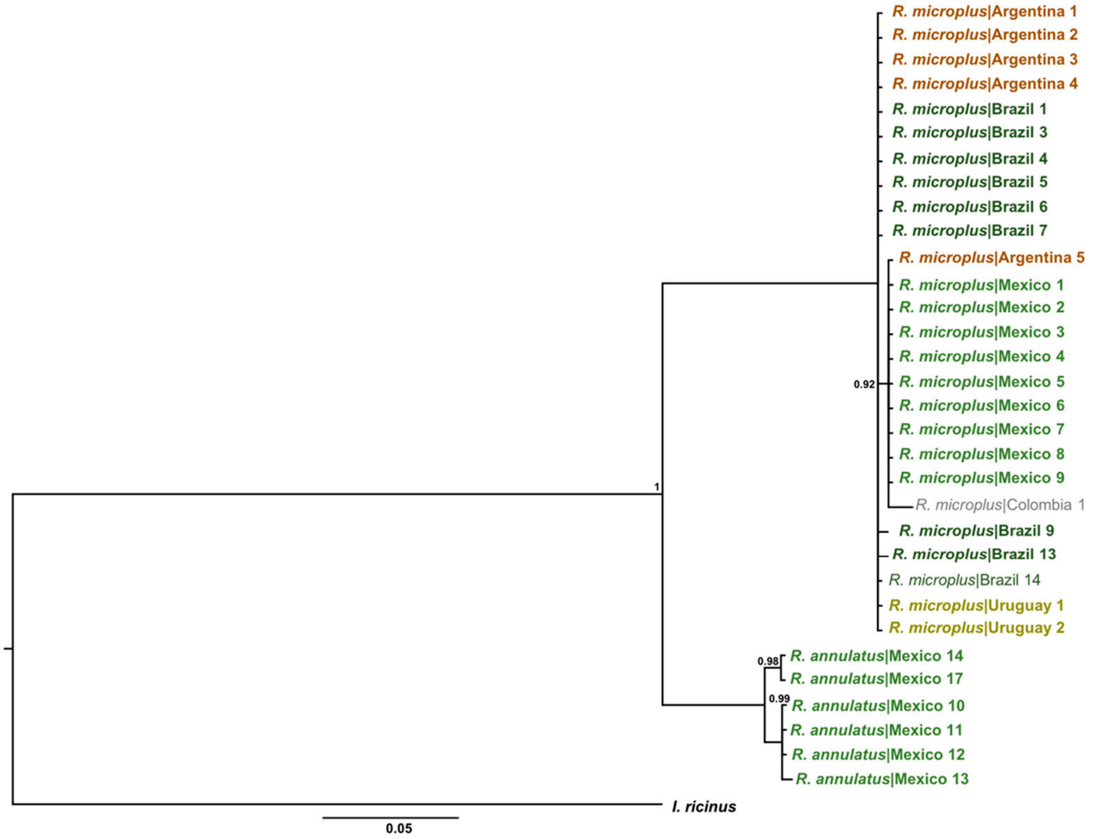

Supplementary File 4. Figure S4.
